# Breast DCE-MRI: lesion classification using dynamic and morphological features by means of a multiple classifier system

**DOI:** 10.1186/s41747-017-0007-4

**Published:** 2017-06-29

**Authors:** Roberta Fusco, Massimiliano Di Marzo, Carlo Sansone, Mario Sansone, Antonella Petrillo

**Affiliations:** 1 Department of Diagnostic Imaging, Radiant and Metabolic Therapy, “Istituto Nazionale Tumori Fondazione Giovanni Pascale—IRCCS”, Via Mariano Semmola, 80131 Naples, Italy; 2 Department of Melanoma Surgical Oncology, “Istituto Nazionale Tumori Fondazione Giovanni Pascale—IRCCS”, Via Mariano Semmola, 80131 Naples, Italy; 3 Department of Electrical Engineering and Information Technologies, University “Federico II” of Naples, Via Claudio 21, 80125 Naples, Italy

**Keywords:** Breast cancer, Dynamic contrast-enhanced MRI, Multiple classifier system, Morphological features, Decision tree, Dynamic features, Bayesian classifier

## Abstract

**Background:**

In breast magnetic resonance imaging (MRI) analysis for lesion detection and classification, radiologists agree that both morphological and dynamic features are important to differentiate benign from malignant lesions. We propose a multiple classifier system (MCS) to classify breast lesions on dynamic contrast-enhanced MRI (DCE-MRI) combining morphological features and dynamic information.

**Methods:**

The proposed MCS combines the results of two classifiers trained with dynamic and morphological features separately. Twenty-six malignant and 22 benign breast lesions, histologically proven, were analysed. The lesions were subdivided into two groups: training set (14 benign and 18 malignant) and testing set (8 benign and 8 malignant). Volumes of interest were extracted both manually and automatically. We initially considered a feature set including 54 morphological features and 98 dynamic features. These were reduced by means of a selection procedure to delete redundant parameters. The performance of each of the two classifiers and of the overall MCS was compared with pathological classification.

**Results:**

We obtained an accuracy of 91.7% on the testing set using automatic segmentation and combining the best classifier for morphological features (decision tree) and for dynamic information (Bayesian classifier). With implementation of the MCS, an increase in accuracy of 12.5% and of 31.3% was obtained compared with the accuracy of the Bayesian classifier tested with dynamic features and with that of the decision tree tested with morphological parameters, respectively.

**Conclusions:**

An MCS can optimise the accuracy for breast lesion classification combining morphological features and dynamic information.

## Key points

- An MCS combined two classifiers trained with morphological and dynamic features.
- A decision tree was used for classifying morphological features.
- A Bayesian classifier was used for classifying dynamic features.
- Combining morphologic and dynamic features, 92% accuracy can be obtained.

## Introduction

Breast cancer is the most common cancer among women in the western world. To date it is the second leading cause of cancer death in women (after lung cancer) and is estimated to cause 15% of cancer deaths [1]. Therefore, screening for early diagnosis of breast cancer is of great interest.

The currently most widespread screening method is X-ray mammography [2]. However, this method is not adequate for young women in the presence of dense breasts. Moreover, detection and characterisation of breast lesions on mammography is difficult because of the lack of functional information.

Dynamic contrast-enhanced magnetic resonance imaging (DCE-MRI) has demonstrated a great potential for screening high-risk women, staging newly diagnosed breast cancers, and assessing therapy effects [1], thanks to the possibility of visualising three-dimensional (3D) high-resolution dynamic (functional) information, not available with mammography or with ultrasound. Therefore DCE-MRI is gaining popularity as an important tool for breast cancer diagnosis [3].

For breast lesion detection and classification using DCE-MRI, radiologists agree that both morphological and dynamic features are important [4–6]. On the one hand, morphological features aim to quantify lesion characteristics well assessed in the breast MRI lexicon [6]: round shape and smooth margin for benign lesions; irregular shape and margins for malignant lesions. On the other hand, dynamic information has shown great potential for quantifying tumour vascularity [6–8]: malignant lesions usually show early enhancement with rapid washout, whereas benign lesions typically show a slow increase followed by persistent enhancement [6].

Recent studies have attempted to take advantage of morphological features and dynamic information: dynamic and morphological data, generally separately, have been used both for segmentation of volume of interests (VOIs) [7–11] and for lesion classification [6–15]. Nie et al. [4] demonstrated that quantitative analysis of the morphology and texture features of breast lesions is feasible. These features could be selected by an artificial neural network for differential diagnosis between breast cancer and benign breast lesions. Agner et al. [14] showed that good performances could be yielded using a probabilistic boosting tree classifier in conjunction with textural kinetic features. However, when the dataset included both textural kinetic and morphologic features, the performance was lower. Zheng et al. [13, 16] investigated the use of a feature set including dynamic, spatial, and morphological features with a linear classifier.

To the best of our knowledge, a multiple classifier system (MCS) for classification of breast lesions using morphological and dynamic features in DCE-MRI has not yet been presented, although the idea of combining multiple classifiers is not new. For example, Keyvanfard et al. [17] proposed an MCS composed of three classifiers that used dynamic features to classify breast lesions in DCE-MRI, but morphological features were not used in their study.

Our aim was to propose an MCS for classifying breast lesions using both morphological and dynamic features on DCE-MRI. As classifiers, we used those best suited for the problem at hand, according to our previous study [18].

## Materials and methods

### Patient selection

Forty-eight women (age 51 ± 18 years, mean ± standard deviation) with pathologically proven benign or malignant lesions were examined retrospectively. All of these subjects had undergone DCE-MRI at our cancer centre. Twenty-six lesions were malignant and 22 were benign. The lesions were subdivided into two groups (Table 1): training set (14 benign and 18 malignant) and testing set (8 benign and 8 malignant). All patients provided informed consent for the use of their data for research purposes. This retrospective study was performed according to regulations issued by our local Institutional Review Board.

**Table 1:** Pathology of lesions included in the training set and in the testing set

Pathology	Training set	Testing set
Malignant		
Invasive ductal	7	4
Invasive lobular	2	1
Invasive ductal lobular	5	2
Ductal carcinoma in situ	4	1
Subtotal	18	8
Benign		
Fibroadenoma	10	5
Ductal hyperplasia	2	1
Fibrocystic dysplasia	1	1
Intraductal papilloma	1	1
Subtotal	14	8
Grand total	32	16

### Data acquisition

The patients underwent imaging with a 1.5-T scanner (Magnetom Symphony; Siemens Medical System, Erlangen, Germany) equipped with a dedicated breast coil with 16 channels. Turbo spin-echo T2-weighted axial images (TR/TE 4000/56 ms; flip angle 180°; field of view 340 × 340 mm^2^; matrix 385 × 385; slice thickness 2 mm; no interslice gap; 56 slices covering the entire breast volume) and turbo spin-echo T1-weighted fat-saturated axial images (TR/TE 564/12 ms; flip angle 90°; field of view 350 × 350 mm^2^; matrix 512 × 256; slice thickness 2 mm; no interslice gap; 80 slices covering the entire breast volume) were acquired for morphological imaging. T1-weighted fast low-angle shot 3D coronal images were acquired (TR/TE 9.8/4.76 ms; flip angle 25°; field of view 330 × 247 mm^2^; matrix 256 × 128; partition thickness 2 mm; no interslice gap; acquisition time 56 s; 80 slices covering the entire breast volume). One series was acquired before and nine series after intravenous injection of 0.1 mmol/kg body weight of a positive paramagnetic contrast material (Gd-DOTA; Dotarem, Guerbet, Roissy CdG Cedex, France). An automatic injection system was used (Spectris Solaris EP MR; MEDRAD, Inc, Indianola, PA, USA) and the injection flow rate was set to 2 ml/s followed by a flush of 10 ml saline solution at the same rate.

#### Manual segmentation

**Fig. 1:**
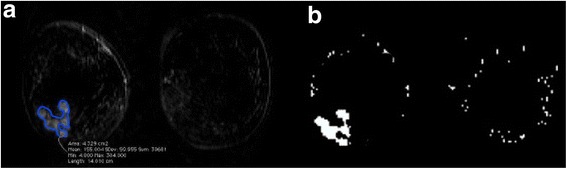
Example of ROI manual segmentation (**a**) and ROI automatic segmentation (**b**) for the same multifocal confluent lesion at the external lower quadrant of the right breast

Regions of interest (ROIs) to cover the entire tumour volume were drawn manually slice by slice, by a radiologist with 22 years of experience in breast MRI, on T1-weighted images obtained by subtracting the unenhanced image from the fifth contrast-enhanced image using the turbo spin-echo T2-weighted images as a guide. For each patient, all of the slices including the lesion were used. The segmentation was performed with OsiriX v.3.8.1 (Fig. 1a).

#### Automatic segmentation

The automatic segmentation algorithm included three steps. The first involves a breast mask extraction by means of an automatic intensity threshold estimation (Otsu thresholding) on the parametric map obtained, considering the sum of intensity differences (SOD) calculated voxel by voxel. This parameter describes the dynamic information of the whole curve and reflects the history of contrast agent enhancement with time:

$$ SO{D}_p= Pr{e}_p+{\displaystyle \sum_{i=1}^T}\left| P o s{t}_p(i)- P o s{t}_p\left( i-1\right)\right| $$

where *SOD*_*p*_ is the SOD for the *p* voxel, *Pre*_*p*_ is the pre-contrast intensity, *Post*_*p*_(*i*) is *i*th post-contrast scan and *T* is the total number of scans.

The second step included hole-filling and leakage removal by means of morphological operators: closing is required to fill the holes on the boundaries of breast mask; filling is required to fill the holes within the breasts; and erosion is required to reduce the dilation obtained by the closing operation [19, 20].

The third step included the extraction of suspicious ROIs. The dynamic features of each voxel were analysed. A voxel was assigned to a suspicious ROI if it satisfies two conditions: the maximum of its normalised time-intensity curve should be greater than 0.3, and the maximum signal intensity should be reached before the end of the scan time. The first condition assures that the voxels within the ROI have a significant contrast agent uptake (thus excluding slow enhancement curves) and the second condition is required for the time-intensity pattern with plateau or wash-out [19, 20]. The choice of the 0.3 threshold was based on the findings by Torricelli et al. [21]. In their study, lesions with an enhancement lower than 50% above the baseline were considered non-tumoral. They also noticed that lowering the threshold to 40% improved the accuracy of diagnosis. We proposed a threshold of 30% in order to reduce the number of false negatives. All procedures were implemented in Matlab R2008a using Image toolbox (Fig. 1b).

### Morphological and dynamic features

According to our previous studies [18–20], we considered a feature set including 54 morphological features and 98 dynamic features. The main categories of morphological features included both two-dimensional (2D) and 3D parameters. The 2D morphological features were: mean, standard deviation, maximum and minimum value of areas; perimeters; complexity; radial length; and spiculation. These were obtained slice by slice and then median values were calculated. The 3D features were circularity, compactness, smoothness, roughness, sphericity, eccentricity, volume, rectangularity, solidity, convexity, curvature, and edge [4, 5, 7]. These were obtained on the entire segmented 3D volume. For dynamic features the main categories included area, maximum intensity ratio, relative enhancement, relative enhancement slope, basal signal, perfusion index, SOD, wash-in, wash-out, and time to peak [3, 7, 11, 13].

The number of morphological and dynamic features was reduced by a feature selection procedure to remove uninformative and unimportant morphological features. To keep the loss of information to a minimum we tested the correlation-based feature selection method and the consistency feature selection method with several searches: the forward search, the backward search, the bidirectional search, the greedy search, and feature ranking methods. The morphological features obtained by the selection procedure were area, eccentricity, compactness, and perimeter. The dynamic features retained by the selection procedure were the sum of intensity difference, basal signal, and relative enhancement slope. Table 2 presents the mathematical formula for both morphological and dynamic features retained by the selection procedure which were used to train and test the classifiers.

**Table 2:** Performance on the testing set obtained by the single classifier

Feature		Formula
Morphological	Area, *A*_*k*_	*n*_*k*_ *dxdy* where *n*_*k*_ is number of voxels in the *k*th slice of the ROI, *d*_*x*_ and *d*_*y*_ represent size of voxels
	Perimeter length, *P*_*k*_	*b*_*k*_ where *b*_*k*_ is number of boundary voxels in the *k*th slice of the ROI
	Compactness in 3D, *COMP*	$\frac{S^2}{V_{3 D}}$ where *S* is the surface and *V* is the volume, defined as follows: $s = {\displaystyle \sum_x}{\displaystyle \sum_y}{\displaystyle \sum_z}{b}_{ROI}\left( x, y, z\right){v}_{size} slic{e}_{th}$ where *v*_*size*_ is the voxel size, *slice*_*th*_ is the slice thickness; and *V* = *n*_*ROI*_ *dxdydz* where *n*_*ROI*_ is total number of voxels in the ROI
	Eccentricity, *ECC*	$\frac{\sqrt{a^2-{b}^2}}{a}$ where *a* is the major axle shaft and *b* is the lower one
Dynamic	Basal signal, BS	Signal intensity before contrast injection
	Relative enhancement, *RE*(*t*_*i*_)	$\frac{SI\left({t}_i\right)- BS}{BS}$ where *t*_*i*_ is the *i*th temporal instant
	Sum of local differences, *SOD*	$SO{D}_p= Pr{e}_p+{\displaystyle \sum_{i=1}^T}\left| P o s{t}_p(i)- P o s{t}_p\left( i-1\right)\right|$

*ROI* region of interest

The classifiers were trained with the morphological and dynamic features extracted by both manual and automatic segmentation to assess whether the automatic procedure could affect the MCS performance.

### VOI classification

**Fig. 2:**
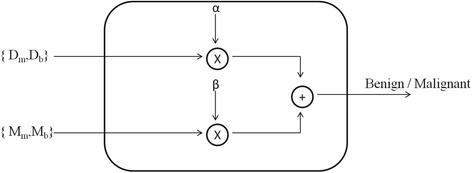
Illustration of the MCS as the combination of a classifier tested with morphological features and a classifier tested with dynamic information. *D*_m_ probability of malignant lesions, *D*_b_ probability of benign lesions, *M*_m_ probability of malignity, *M*_b_ probability of benignity, *α* and *β* multiplicative coefficients (*α* + *β* = 1)

The proposed MCS combines the results of two classifiers trained separately with dynamic and morphological features, respectively (Fig. 2). In particular, the weighted sum of probability of malignancy and the probability of benignancy of the two chosen classifiers as proposed by De Santo et al. [22] were considered.

The choice of the proposed classifier was based on a previous study [18], where a decision tree (collaborative filtering = 0.25; unpruned = true) and a Bayesian classifier (Kernel estimator = false) gave us the best results when trained on morphological and dynamic features, respectively. We estimated the classifier parameters using the training data and the subset of features obtained by feature selection methods varying classifier parameter values and evaluating the accuracy. A 10-fold cross-validation procedure was chosen to train the classifiers.

Then, in order to combine the results of the two classifiers, each suspicious voxel within the VOI was first classified as benign or malignant based on dynamic information. The whole VOI was then classified as malignant if the number of malignant voxels (*n*_m_) within the VOI was higher than benign voxels (*n*_b_) within the same VOI.

The probability of malignant lesions (*D*_m_) and the probability of benign lesions (*D*_b_) were calculated as follows:

$$ {D}_{\mathrm{m}}={n}_{\mathrm{m}}/ N $$

$$ {D}_{\mathrm{b}}={n}_{\mathrm{b}}/ N $$

where *N* is the total number of voxels in the lesion.

Morphological features were instead calculated for the whole VOI and used to classify the lesion into malignant and benign. In this case, the probability of malignancy and the probability of benignancy were *M*_m_ and *M*_b_, respectively.

Finally, the VOI was classified as malignant if *αD*_m_ + *βM*_m_ *> αD*_b_ + *βM*_b_, where *α* and *β* were multiplicative coefficients (*α* + *β* = 1) which must be suitably chosen in order to maximise the accuracy (Fig. 2).

We estimated *α* and *β* coefficients using the test set lesions group, the subset of features retained by feature selection methods, and the optimised classifiers obtained by training data set analysis and evaluating the accuracy of the MCS. A leave-one-out cross-validation procedure was chosen as suggested by Torricelli et al. [21].

Machine learning analysis was performed using Weka open source software (http://www.cs.waikato.ac.nz/ml/weka/).

### Statistical analysis

Sensitivity, specificity, positive and negative predictive values, and accuracy were reported for each of the two classifiers and for the MCS in the case of both the manual and the automatic segmentation procedure. The McNemar test was used to assess differences between manual and automatic segmentation. Calculations were performed using the Statistic Toolbox of Matlab R2008a. Values of *p* lower than < 0.05 was considered significant.

## Results

Table 2 presents the morphological and dynamic features retained by the feature selection procedure and that were used to train the individual classifier. Table 3 presents the results obtained on the testing set by each of the two classifiers using dynamic or morphological features, both for manual and automatic segmentation. We observed a significant difference between the performances of classifiers when manual or automatic segmentation was performed.

**Table 3:** Performance obtained by the proposed methods on the testing set

Classifier	Segmentation	Sensitivity (%)	Specificity (%)	PPV (%)	NPV (%)	Accuracy (%)	*p* value^a^
Bayesian classifier using dynamic features	Manual	92.3 (24/26)	81.8 (18/22)	85.7 (24/28)	90.0 (18/20)	87.5	0.04
	Automatic	88.5 (23/26)	68.2 (15/22)	76.7 (23/30)	83.3 (15/18)	79.2	
Decision tree classifier using morphological features	Manual	92.3 (24/26)	77.3 (17/22)	82.8 (24/29)	89.5 (17/19)	85.4	0.02
	Automatic	76.9 (20/26)	40.9 (9/22)	60.6 (20/33)	60.0 (9/15)	60.4	

*PPV* positive predictive value, *NPV* negative predictive value

^a^McNemar test

**Fig. 3:**
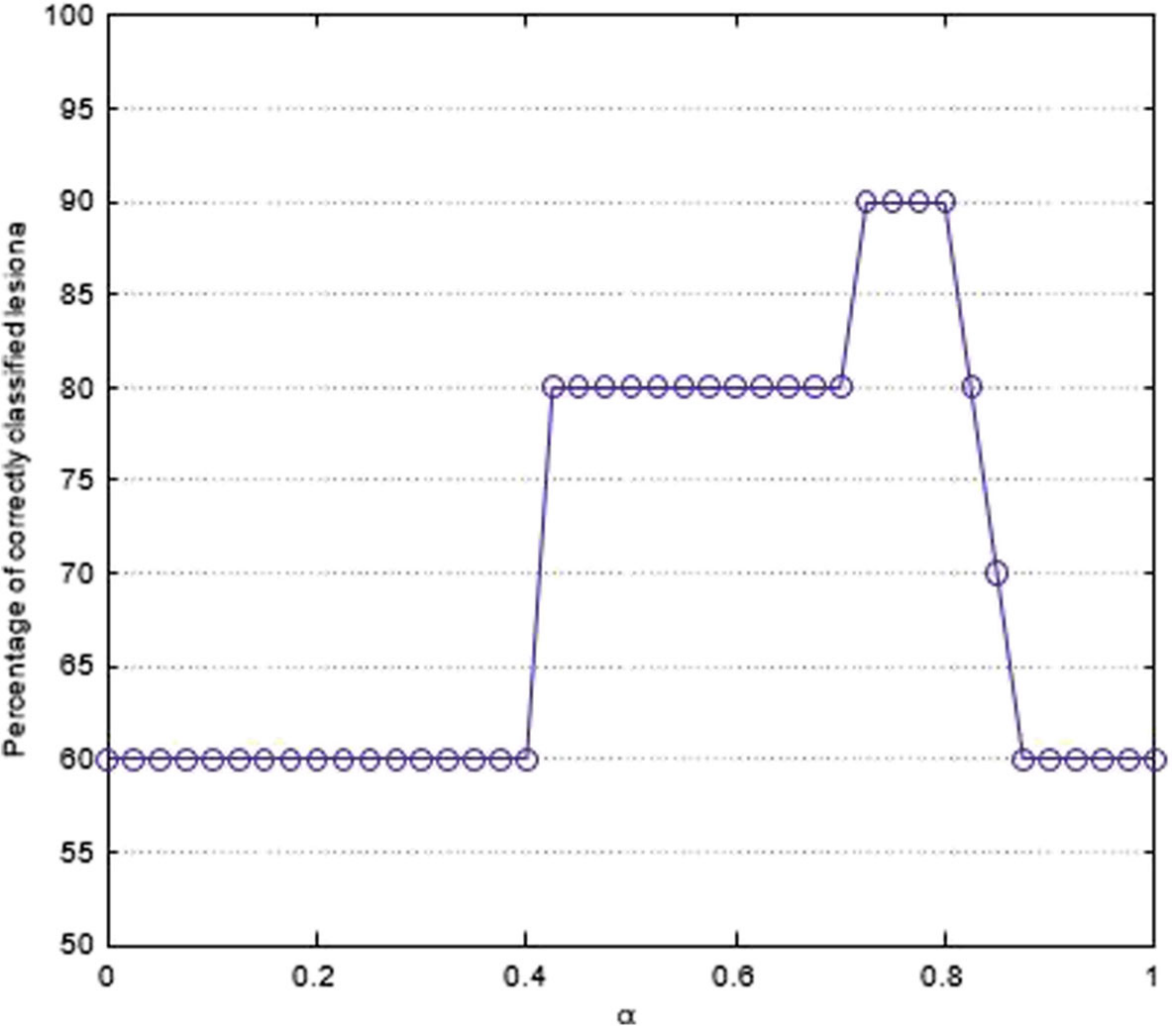
Percentage of correctly classified lesions by the proposed MCS versus the coefficient *α*

As far as the choice of optimal values for *α* and *β* is concerned, we report in Fig. 3 the percentage of correctly classified lesions versus *α*. The best compromise among sensitivity (92.3%), specificity (90.9%), positive predictive value (92.3%), negative predictive value (90.9%), and overall accuracy (91.7%) resulted in *α* = 0*.*75 with only two false negatives and two false positives. With MCS implementation, an increase in accuracy of 12.5% and of 31.3% was obtained when comparing the Bayesian classifier using dynamic features with the decision tree using morphological features, respectively.

## Discussion

The aim of this study was to propose an MCS to classify breast lesions on DCE-MRI. The proposed MCS combines the results of two classifiers trained and tested using dynamic and morphological features separately.

It is well known that training machine learning classifiers with a large number of features can lead to classifier overfitting, reducing the generalisation capabilities of the classifiers and slowing down the training process. As a consequence, a selection procedure was performed. The features retained by the selection procedure (area, eccentricity, compactness, and perimeter as morphological features; and SOD, basal signal, and relative enhancement slope as dynamic features) were used to assess the performance of the single classifier and of the MCS.

In our previous studies [18, 20] we analysed the performance of several classifiers (multilayer perceptron, support vector machine, Bayes classifier decision tree), in conjunction with dynamic and morphological features, separately using a ROI manual selection slice by slice. In this study we compared the results of accuracy to differentiate benign from malignant lesions using both manual and automatic segmentation procedures. We found a significant difference between the performances of classifiers when manual or automatic segmentation was used. Therefore, an automatic VOI segmentation procedure could affect the overall performance. However, our results proved that, although a single classifier trained separately with dynamic and morphological features achieves a low overall accuracy, the MCS could optimise the correct classification rate: dynamic features with the decision tree gave 60% accuracy, morphological features with the Bayes classifier gave 80% accuracy, and the MCS gave 91.7% accuracy. Overall accuracy of the MCS is comparable with the accuracy of each of the two classifiers when a manual segmentation procedure was performed. These results are interesting because ROI manual selection performed slice by slice is time consuming and operator dependent.

The findings of this study are in line with recent literature [23, 24]. In fact, Wedegärtner et al. [23] reported a sensitivity of 83% using morphological features (irregular lesion contour) and an area under the receiver operating characteristic curve of 0.9 for 62 breast lesions without automatic classification. Tzacheva et al. [15] reported a sensitivity of 90%, a specificity of 91%, and an accuracy of 91% using morphological features and a multilayer perceptron classifier on 14 breast lesions. However, these authors did not use an automatic segmentation step. Zheng et al. [16] reported a sensitivity of 95% using a combination of temporal, spatial, and morphological attributes and a linear classifier for 31 subjects, but even in this study the segmentation step was not completely automatic. Thus, the novelty of our study is the use of a completely automatic MCS.

Further investigation is required for an optimal choice of *α* and *β* because the specific value could affect the overall accuracy of the system (see Fig. 3). It is worth noting that there is an interval of values (0.7, 0.8) in which high performance can be obtained.

An important limitation of this study is that the classifier was trained and tested only with a small number of subjects. As a consequence, our preliminary results need to be confirmed on a larger number of patients. Moreover, manual segmentation could be done by multiple readers to assess inter-observer variability. Finally, a combination of morphological, dynamic, and also texture features could be performed.

In conclusion, we proposed an MCS to classify breast lesions on DCE-MRI combining the results of two classifiers tested with morphological features and dynamic information separately. The MCS could optimise the correct classification rate, reaching an accuracy of 91.7%.
